# Diet and identity: being a good parent in the face of contradictions presented by the ketogenic diet

**DOI:** 10.1111/1467-9566.12330

**Published:** 2015-09-18

**Authors:** Michelle Webster, Jonathan Gabe

**Affiliations:** ^1^ Centre for Criminology and SociologySchool of LawRoyal HollowayUniversity of LondonUK

**Keywords:** diet, parenting/parents, food, epilepsy, identity

## Abstract

The ketogenic diet is a high‐fat diet used to treat drug‐resistant childhood epilepsy. Given that negative meanings tend to be attached to fatty foods and children's food consumption is seen to be the responsibility of parents, the ketogenic diet may be problematic for parenting identity. This article draws upon in‐depth semi‐structured interviews with 12 parents from 10 families that have a child whose epilepsy is being treated with the ketogenic diet. The main focus of the article is the meanings these parents attached to foods and how they were drawn upon or altered to overcome some of the contradictions presented by the diet. It will be argued that the diet was medicalised and parents came to view food as medicine. When viewing food in this way, negative associations with fat were reversed. Furthermore, parents also used food as a symbol of inclusion and prioritised portion size or the child's enjoyment of food in order to use food as a symbol of love. In turn this enabled parents to feel they were being good parents. Overall, it seems that diet can be medicalised and the identity of the good parent maintained if dietary treatment is successful.

## Introduction

The ketogenic diet is a high‐fat, low‐carbohydrate diet used to treat drug‐resistant childhood epilepsy. It was originally introduced as a treatment for epilepsy in 1921 but use declined with the introduction of the medication diphenylhydantoin in 1938 (Wheless 2008). However, over the past 15–20 years there has been a resurgence of interest in the diet, and its popularity has increased in the USA and the UK (Wheless 2008). Between 2000 and 2007 the number of children being treated with the ketogenic diet in the UK increased by 50 per cent, bringing the total in 2007 to 152 (Lord and Magrath 2010). Recently a leading dietician has claimed that the number of children receiving dietary treatment for epilepsy in March 2014 was 536, according to evidence from the caseload database for UK centres (personal communication). This figure suggests that the number of children on the diet has continued to increase since 2007.

Although the exact mechanisms of the diet are still unknown (Neal *et al*. 2008), it causes the body to go into ketosis^1^ and controls seizures by mimicking the metabolic effects of starvation (Cross 2012). The first randomised controlled trial, conducted by Neal *et al*. (2008), demonstrated the diet's efficacy, as it was found that after 3 months 38 per cent of children on the diet had experienced a 50 per cent reduction in seizures compared with only 6 per cent of the control group who continued to be treated with medications alone.

This article draws on the experiences of families using three different forms of dietary treatment: the classical ketogenic diet, the medium‐chain triglyceride (MCT) diet and the modified Atkins diet (MAD). Although the MAD is not medically defined as a ketogenic diet, for the purposes of this article it will be referred to as such due to its high fat content. Furthermore, the parents employing this diet considered it to be a form of ketogenic treatment, as they reported that their child was in a state of ketosis when using the diet. The ratio of fat to protein and carbohydrate on the diet varies between 2:1 and 4:1, meaning that up to 80 per cent of calories are received in the form of fat (Cross 2012). The MAD and classical diets rely on large amounts of butter and cream for their high fat content (Ferrie *et al*. 2012) while the MCT diet uses MCT oil. Other than the type of fat that each diet uses, the main difference between the diets is that protein is not limited on the MAD. As the findings discussed in this article relate to all three diets, with no major discrepancies found between them, they will be referred to collectively from now on.

Due to the nature of the ketogenic diet, foods are selected primarily for their nutritional content. Although individuals not on a specific diet may give consideration to nutritional content when making food choices, this is not the principal way in which foods are usually selected (Beardsworth and Keil 1997); rather, food choice is largely a result of the meanings attached to foods and the social context in which they are eaten (Delormier *et al*. 2009, DeVault 1991, Wills *et al*. 2011). Indeed, there are norms related to when, where and with whom we eat (Counihan 1999, DeVault 1991, James *et al*. 2009). As will be outlined below, the ketogenic diet contradicts a number of these norms.

While the number of children being treated with the diet has continued to rise, little is known about parents’ experiences of implementing it. The purpose of this article is therefore to examine how parents managed their identities despite the contradictions raised by the diet.

## Food, identity and parenting

The meanings associated with different foodstuffs influence food consumption. People often use ideas of good and bad foods to distinguish between those that are perceived to be beneficial to one's health and those that are seen to be detrimental (Counihan 1999, Lupton 2005). Foods that are high in sugar, and particularly those with a high‐fat content, are viewed as bad foods because they have been linked to the development of a number of chronic health conditions (Counihan 1999, Lupton and Chapman 1995, Lupton 1996, 2005). Health campaigns stretching back to the 1970s have recommended that people should reduce their fat intake (Beardsworth and Keil 1997) and these campaigns have become even more prevalent in recent years (Blank *et al*. 2009). The high fat content of the ketogenic diet therefore contradicts assumptions about the type of foods that should be eaten.

The meanings attached to foods not only influence food choice but also identity construction. People have been found to make judgements about themselves, and even more about others, based on the types of food they eat; those who consume bad foods with a high‐fat content are sometimes seen to be bad people who lack self‐control (Lupton 1996, 2005, Saguy 2013). Similarly, judgements are often made about parents based on the types of food they feed their children (Dixon and Banwell 2004, Saguy 2013). Indeed, parents often comment that they feel responsible for the provision of healthy meals (Cook 2009a, 2009b, Stapleton and Keenan 2009). Based on this literature, there is a potential for parents to feel guilty if they believe they are not feeding their children a diet that is currently deemed to be healthy.

Despite parents feeling responsible for providing children with healthy food, over the past two decades it has been claimed that children's influence over their food consumption has generally increased (Dixon and Banwell 2004). This has been accompanied by a shift in parenting philosophy, whereby parents now feel that children should be able to express their own opinions and food should not be forced upon them (Coveney 2006, Dixon and Banwell 2004). It appears that this philosophy is particularly prevalent among working‐class families, where it has been found that children's food choices are readily accepted (Backett‐Milburn *et al*. 2006) and the development of autonomy is encouraged (Wills *et al*. 2011). The findings of these studies illustrate that the meanings attached to food and eating vary among different classes due to the habitus – acquired dispositions and tastes – of each group (Bourdieu 1984, Wills *et al*. 2011). It may be the case that the ketogenic diet limits parents’ ability to satisfy their child's food requests because of the nature of the diet. Furthermore, as fat has a high calorific content, the portion sizes of ketogenic meals are often smaller than people are used to, meaning that parents may feel they are not providing their child with enough food. Therefore, it is possible that the nature of the diet may cause parents, particularly working‐class parents, to feel conflicted between implementing the diet and fulfilling their child's food desires.

Food has cultural significance not only for people on an individual level but also for the family as a whole. This is because love and care are displayed through feeding others and sharing food (DeVault 1991, Lupton 1996, Warin *et al*. 2008). Although it is acknowledged that family meals can be a site of conflict (Wilk 2010), this cultural ideal is still aspired to in many families, as parents see it as a social event that brings family members together (James *et al*. 2009, Stapleton and Keenan 2009). While there is no clear, agreed upon definition of what a family meal is, a common feature is all family members eating together and, traditionally, everyone present eating the same meal (Blake *et al*. 2009, Gallegos *et al*. 2011). There is scope for the ketogenic diet to contradict this traditional notion of a family meal because family members are likely to be eating different kinds of food; however, as individuals’ interpretations of the family meal vary, whether parents feel the diet does in fact contradict this norm is unknown.

Previous discussions on parenting a child with epilepsy are dated and draw on adults’ recollections of their childhoods with the condition, rather than on parents’ views (Scambler and Hopkins 1988, Schneider and Conrad 1983). However, research focusing on families’ responses to the use of diet by a family member for other medical reasons indicates that these other family members often assimilate dietary changes by adjusting their own food consumption, either for practical reasons or to normalise dietary alterations (Gregory 2005, Pitchforth *et al*. 2011). This response has been found in families where one family member had diabetes (Kelleher 1988, Maclean 1991), a nut allergy (Pitchforth *et al*. 2011), coeliac disease or coronary heart disease (Gregory 2005). An alternative response, found in families where a child had coeliac disease, was to demedicalise the diet by treating food consumption as ‘a matter of choice rather than prescription’ (Veen *et al*. 2013: 592).

To date, the ketogenic diet has only been studied by biological scientists. Consequently, despite the rise in the number of children being treated with the diet, little is known about how parents manage their identities in relation to the contradictions the diet presents. Furthermore, the restrictive nature of the diet means that it differs somewhat from diets that have previously been studied. As a result, there is scope to add to the current literature on the use of dietary treatments within the family. The purpose of this article is, therefore, to broaden understanding and provide an insight into how parents manage their identities despite the contradictions raised by the diet.

## Methodology

A qualitative approach was employed to explore, in detail, parents’ experiences of using the diet to treat their children's epilepsy. During 2013 the research was advertised through three UK‐based charities: Epilepsy Action, which supports individuals with epilepsy and their families, and two charities that support families using the ketogenic diet – the Daisy Garland and Matthew's Friends. The charities placed adverts provided by the first author on their websites, online forums, social media pages, and in their newsletters.

Parents from 15 families came forward as a result of the adverts and in‐depth semi‐structured interviews were carried out with 12 parents from 10 of those families. One parent decided not to participate because she had just begun implementing the diet and felt she could not fit in an interview. It is unknown why parents from the other four families chose not to partake. Although the sample was small, additional participants were not recruited as theoretical saturation had been reached. Roughly equal numbers of parents were recruited per charity and they had varying levels of involvement with the charities and other parents using the diet. Consequently, it is unlikely that the meanings parents attached to foods were learnt entirely as a result of such interactions.

All families were two‐parent families and had between two and four children. The children on the diet consisted of four girls and six boys aged between 3 and 10 years. Seven of the children were on the classical version of the diet, two were on the MCT diet and one was on the MAD. Overall, the data presented below comprise the views of 10 mothers and two fathers (where both parents from one family participated they were interviewed together; consequently, two were joint interviews and eight were individual interviews). The great majority of participants were White British or White European, with one parent being Asian (foreign‐born). Although the families were from a range of socioeconomic groups, most were in the top quartile of earners.

Six interviews were conducted face‐to‐face, two were phone interviews, one was conducted via Skype and one was an e‐mail interview. It was not possible to conduct all of the interviews face‐to‐face due to the location of some of the participants; although UK‐based charities advertised the research, two parents from outside the UK who used one of the charity's online forums asked to participate. Those who were interviewed face‐to‐face were all from mainland UK, and those who were interviewed using other methods were from non‐mainland UK, Eastern Europe and Western Europe (all the interviews were conducted in English). Additionally, most interviews were conducted with participants in their own homes, with the exception of one, which was carried out in a café at the participant's request.

One limitation of the phone and Skype (without video) interviews was that the researcher was unable to use non‐verbal cues and, therefore, at times it was difficult to know whether the participant had finished speaking or whether they were pausing to think. However, when comparing the data, no disparity existed in terms of richness between the interviews conducted in person and those conducted using alternative means. It is acknowledged that all statements participants made were a product of the social space in which they were created (Power 2004). However, it was still felt that in‐depth interviews were the most appropriate research technique as they allowed for an in‐depth exploration of an under‐researched area. Furthermore, the presence of both parents in two of the interviews undoubtedly shaped the data as there were sometimes disagreements, but this did provide an insight into the perspectives of both parents.

The interviews lasted between 1–2 hours and focused on the child's daily food consumption and parents’ daily routine in relation to implementing the diet. Parents often gave very rich answers and used stories to illustrate their points. Consequently, parents also mentioned their child's food preferences, its preparation time, cost, managing the diet on special occasions, difficulties associated with implementing the diet and how they fitted the diet into their daily lives, as well as others’ reactions to the diet. If the participants themselves did not raise these topics they were probed about them.

All interviews were audio‐recorded and transcribed verbatim*,* with the exception of the e‐mail interview. The data were then coded using NVivo and analysed using a constructivist grounded theory approach (Charmaz 2006). In contrast to Glaser and Strauss’ (1999) grounded theory, a literature review was conducted prior to carrying out the interviews in order to gain an understanding of previous research on similar topics. But, in accordance with Glaser and Strauss (1999), themes were developed using the constant comparative method throughout the data collection phase, emerging themes were drawn upon in later interviews to fill gaps in the analysis, and participants were recruited until categories became saturated. Importantly, a constructivist grounded theory approach was adopted because it is underpinned by the belief that data and theories are constructed. From this standpoint, data are jointly constructed by the participant and researcher, and the theory that results from the analysis is an ‘*interpretive* portrayal of the social world, not an exact picture of it’ (Charmaz 2006: 10, original emphasis).

Ethical approval was granted by the Centre for Criminology and Sociology's departmental ethics committee at Royal Holloway, University of London, prior to beginning data collection. In line with this approval, participants and their family members are referred to using pseudonyms to maintain their anonymity.

Below, we report the findings from the study. We start by clarifying the contradictions posed by the diet and defining the good parent as a concept derived from parents’ discussions. Following this, four techniques used by parents to manage their identities are outlined. Two of these techniques were particular to the ketogenic diet (food as medicine and fat as good), while the other two have been more commonly discussed in the literature on food and parenting (food as a symbol of inclusion and food as a symbol of love).

## Findings

The main contradictions presented by the diet were that it was high‐fat, portion sizes were small and the child was often unable to eat the same food as their family members and peers, thereby contradicting the feeding norms associated with parenting, described above. Parents drew on the concept of the good parent to overcome these contradictions and to help manage their identities. It is acknowledged that notions of good parenting vary between groups and change over time (Lee *et al*. 2014). Here, it is the participants’ perceptions of good parenting that are presented. Parents felt they could overcome some of the contradictions outlined above by altering the meanings attached to foods or by selecting foods that adhered to one or more of the food norms related to parenting (for example, providing adequate portion sizes and ensuring children enjoy their food). The following two sections describe two interrelated adjusted meanings associated with the child's food.

### Food as medicine

The first way parents overcame some of the contradictions presented by the diet is described by Naomi in the following quotation:

I do sometimes think ‘Oh, I should really give her more variety’. But you kind of have to start thinking of food as medicine. You don't have to have too much emotion attached to it.

DeVault (1991) notes that norms related to feeding others tend to be referred to more directly when people feel that they are unable to follow these norms; a feature which was common in these parents’ discussions about the diet. Indeed, this is what Naomi is doing here when she acknowledges the importance placed on consuming a varied diet. However, Naomi then describes how she overcame this contradiction – by viewing food as medicine. It is acknowledged that medicines are not always viewed positively (Britten 2008); however, in this instance, parents used this terminology to express the beneficial impact this treatment was having on their child.

This view of food as medicine is in stark contrast to Veen *et al*.’s (2013) research on families with a child with coeliac disease, where it was found that dietary alterations were demedicalised. Instead, parents in this study drew on the medical model – the dominant approach to illness in western society that assumes an underlying pathological cause of illness and gives authority, regarding the diagnosis and treatment of illness, to those in the medical profession (Bury 2013). Drawing on this model enabled parents to view food functionally in the sense that they saw the entirety of the child's food consumption as a treatment for their condition. Naomi described how, by viewing food in this way, some of the meanings and norms attached to food became irrelevant. Indeed, when these norms were stripped away, the child's food could be thought of purely in terms of the benefit this dietary treatment was having. All these parents had seen a reduction in their child's seizures, many described other benefits such as increased alertness and some had been able to reduce the child's medication and felt this had resulted in fewer side‐effects. These benefits undoubtedly contributed to the good parent identity as the parents were providing an effective treatment for their child's epilepsy.

When speaking about creating meals, parents tended to talk about the child's food in a scientific way. They often spoke about the child's prescription – the amount of fat, protein and carbohydrate each meal had to contain – again linking to the idea of food as medicine. Below are quotations relating to the three different diets that illustrate the way in which food was spoken about. The type of diet being referred to is given in brackets at the end of each statement.

Paul He's on a 4:1 ratio. (Classical)

Hannah We've just tweaked the diet again, so at the minute it's 29 fat [points] and we're up to 9 carbs. (MAD)

Kelly When we started he was on 16 exchanges a day. And … 81 units of MCT. And … six of those 16 [exchanges] needed to be protein sources. (MCT)

The classical version of the diet was spoken about using ratios of fat to protein and carbohydrate, and the MAD and MCT diet were thought of in terms of exchanges or points relating to each food type (fat, protein and carbohydrate). Chowdhury *et al*. (2000) note that, unlike nutritionists, lay people rarely classify foods into these types; however, these parents regularly talked about food in this way. It is likely that speaking about food in these terms is a form of the ‘cultural health capital’ (Shim 2010) that the parents had developed through their regular interactions with dieticians. However, this did not appear to be class specific, as the two working‐class parents also drew on this discourse.

The extent to which the parents were able to view food as medicine varied between families, but for many it was a practical way of overcoming some of the contradictions presented by the diet. Parents who were able to view food primarily as medicine were those who had been using the diet longest and those whose children were young or had comorbidities or learning difficulties associated with their epilepsy. Some of the children's learning difficulties resulted in them not being particularly interested in food; therefore, parents did not feel they were depriving their child of foodstuffs they wanted. Equally, children who were young often did not compare their food to others’ food consumption; this meant they did not feel they were missing out, which again meant parents did not regularly feel guilty when implementing the diet.

It has been noted that viewing food as medicine is common in many cultures (Helman 2007). In the UK the health‐promoting properties of vitamins began being advertised during the 1920s and influenced the way in which the nation viewed food (Horrocks 1995). This change in attitudes resulted in certain foods being seen to have medicinal value. Although the perspective of parents in this study is similar in that they also linked food and health, it differs because rather than specific foods, all the foods the child consumed were seen to have medicinal value.

As a result of coming to view the child's food as medicine and by focusing on the purposeful aspect of the diet, a further new meaning was attached to the child's food – fat came to be seen as good.

### Fat as good

Interestingly, despite the negative meanings that are normally attached to fat (Counihan 1999, Lupton and Chapman 1995, Lupton 1996, 2005, Saguy 2013) and the importance placed on feeding children a healthy diet (Cook 2009a, 2009b, Stapleton and Keenan 2009), the high‐fat content of the diet was not something that parents found problematic. This can be explained by the fact that parents had started to view food as medicine and were focusing on the benefits this ‘medicine’ was having for their child; in so doing they were able to reverse the negative meanings attached to fat and fatty foods. For example, when speaking about choosing different products Jane said, ‘the more fat the better’. This is not a statement a parent would typically make in relation to their child's food consumption. However, it was echoed by a number of parents who said that they checked product labels and sought out those with the highest fat content.

Similarly, a number of parents spoke to their children about ‘the magic diet’. In the extract below, Jessica is describing how the staff at her son's new school told him not to drink the oil that was left when he had finished his salad:

He even told me that the other day he wanted to drink his salad sauce and then they said ‘no, no, no. Just leave it’. So I'm going to have to tell them tomorrow ‘no, that's the magic ingredient in that sauce’.

Again the term magic is used in relation to the diet and, most importantly, the fat content of the food. This phrase was most commonly used to emphasise the positive impact of the diet, and particularly the high‐fat content, to children and those unfamiliar with the diet. However, it also illustrates the positivity with which parents viewed the diet as it was felt to have improved their child's quality of life to a far greater extent than other previous treatments. Furthermore, the above extract illustrates that views of fat had been altered in these families and that there was perceived to be a need to educate others appropriately on the importance of administering the child's ‘medication’ correctly.

This positive view of fat helps to explain why parents did not find implementing a high‐fat diet emotionally troubling; by seeing fat as good they could view themselves as good parents because they were feeding their child good foods. Indeed, a number of parents described the diet as healthy:

Ellen I *really* do think it's a healthy diet. You're burning off fat quickly and you're getting the right amount of calories. It's weighed, it's to the gram. And she's getting a whole range. And she looks perfectly healthy on it.

The above discussion has illustrated that families using the ketogenic diet came to see food as medicine and fat as good. However, some meanings usually attached to foods remained unchanged and the child's food was adjusted in accordance with these norms. Although it was shown above that by viewing food as medicine parents were sometimes able to see food in a functional way, their emotions were often still linked to feeding their child.

### Food as a symbol of inclusion

The third way parents were able to construct themselves as good parents was to use food as a symbol of inclusion. It has previously been argued that food is an important feature of family life as it symbolises cohesion or inclusion in a particular group (James *et al*. 2009, Stapleton and Keenan 2009). This meaning was consistently drawn upon by parents when discussing what their child ate, when and with whom.

Special occasions or family events were common situations for parents to use food in this way. For example, Jane organised a ketogenic birthday party for her son where all the children were eating essentially the same foods. In other families, rather than being the same, foods just had to have a similar appearance. For instance, Hannah explained:

We went to my Step‐mum's for Easter and she did a ketogenic meal … . It is egg whites all beaten up and that's his. And she puts some little berries on it … and that's his pudding. So then everybody else had pavlova so it all looked the same colour … . And then the dinner itself, some of the kids didn't have starters so that wasn't a big deal. And then the main meal, his was all cut up and mixed up together, so was Alfie's [sibling] so that's just how Granny did it for both of them.

As Hannah notes, using food in this way was intended to include the child in events surrounding food in the same way as their siblings and peers. Rather than foods just having a similar appearance, a further way of including children was to create ketogenic alternatives to the foods being consumed by others. Common alternatives included sweets, chocolate, cake and other foods that are usually seen as treats. For example, Hashani said:

Lately, because it's been the summer and her sister has been having ice cream and lollies from the freezer I've started making her keto lollies, which she really likes. And again, they'll sit and have those together, which is really nice.

Significantly, as in Hashani's statement above, these alternatives tended to be given at times when siblings or peers were eating these foods. This meant that parents could feel they were treating children equally and that the child on the diet was included.

In some instances, parents would make ketogenic alternatives for entire meals. Two parents who went to great efforts to create alternatives of many meals were Paul and Alison. They explained that the diet had initially challenged their parenting identities, as they had felt guilty eating in front of their son:

Alison I think at first it was quite, it was the guilt. You know, we're sat here eating a Yorkshire pudding and a roast potato …

Paul With these big beady eyes looking at you and you're thinking ‘I can't cope with this’.

Alison Yeah. At the beginning we never ate together, did we?

Paul No, no.

Alison Because we felt too guilty knowing that he couldn't have …

Paul What we were having.

Alison Things that he loved. He loves food.

However, they then explained how they felt it was important for them to move past feeling guilty because it was vital that meal times were social times. They described how they restored their good parent identity by creating ketogenic alternatives of meals so that their son felt included. On the evening of the interview Connor, who was on the diet, and his older brother Joel ate Bolognese together. Joel had pasta and Bolognese and Connor had meat and vegetables in a butter and tomato sauce. Importantly for this family, they referred to both meals as Bolognese. As well as parents creating ketogenic alternatives, some also modified their food consumption and ate certain meals more frequently so the child on the diet could eat something similar.

Not all parents used ketogenic alternatives to encourage their child to feel included; however, most of them did ensure the child ate at the same time and in the same place as others. Like Paul and Alison, many parents felt meal times should be sociable events and that children should never eat alone. For instance, although Hashani's daughter often ate before the rest of her family to ensure her meals were evenly spaced, she was still included in the evening family meal:

Hashani We did try giving her food at the same time as us. She just plays with it and throws it around. So what we do, we'll give her some salad or some vegetables so that she's at the table. Or even just an empty plate with a knife and fork. [Laughs] . … Yeah, she'll sit with us.

Therefore, many parents still felt they were able to have family meals, even if they were eating different foods, because they could eat together. Alternatively, as in the example above, just the presence of all family members was enough for some to consider the meal a social event that included everyone. Additionally, parents’ prioritisation of family meals reinforces the argument that parents still attach much significance to the family meal (James *et al*. 2009, Stapleton and Keenan 2009). Importantly, parents could view themselves as good parents because they were able to uphold this norm while implementing the diet.

However, parents did alter some norms surrounding eating practices. For example, family members did not share their food with the child on the diet. Equally, these children were taught not to share their food:

Peter There were little games that we played …. We made her pancakes at breakfast and said ‘Oh, that looks lovely. Can I have some?’ ‘No. It's mine’. So that possessiveness about her diet … .

Ellen It was *her* diet.

Peter It was her diet. No one else could eat it.

Despite this alteration to eating practices, parents were still able to use food in other ways to ensure the child was included. A further meaning that was not changed as a result of using the diet was that food was seen as a symbol of love.

### Food as a symbol of love

Many of the decisions parents made in relation to their child's food involved prioritising either the child's enjoyment of food or larger portion sizes, both of which were ways of giving to children and using food to symbolise love. Consequently, the parents could feel they were being good parents by providing for their children in these ways.

There was no specific hierarchy between norms relating to portion size and the child's enjoyment of food; instead parents drew on these norms in different instances. For example, in the extract below, Kelly explains that she had made her son a ketogenic birthday cake but would not be doing so again in the future because he was allowed such a small portion:

He's always loved chocolate cake so we thought for his birthday last year we'd try that, and he did eat it but the amount he could have of the cake was, I don't know, the size of a small matchbox. It just was tiny… it was about two or three exchanges for this tiny bit. Whereas, I said to my husband that he loves pears, he loves strawberries, you can get tons of pear and tons of strawberries for that. And you could put some cream on it. You could put sweetener in the cream.

Kelly initially drew on norms related to showing love by providing children with foods they enjoy (Lupton 1996). However, she justified her choice of not repeating this in future and showed her love for her son by prioritising portion size instead. This discursive reasoning is similar to the reconciliation of repertoires described by Will and Weiner (2014). In their research on cholesterol‐lowering foods they found that people drew on the repertoires of health, pleasure, sociality and pragmatism when speaking about their food choices. Drawing on these categories, here the discussion of norms relating to portion size and the child's enjoyment of food both relate to the repertoire of pleasure; however, they appear to constitute separate and distinct repertoires in this context.

Small portion sizes were something that parents often tried to compensate for in different ways. For example, Jessica said, ‘we have these tiny dishes that make it look like a lot’. And, like Kelly above, many parents talked about how they would choose one food over another because it appeared to be a larger portion. These examples show that the parents found small portion sizes challenging to their parenting identity and had found different ways to overcome the problem, enabling them to feel they were being good parents.

However, portion size was not always given priority when the parents made choices associated with the child's food consumption. In the extract below Kelly is explaining why she opted for her son to go on the MCT diet over the classical version of the diet:

In general as he's got a bit older he enjoys food. So I didn't want to take that away from him and I wanted to keep him eating things that he enjoyed eating, even if it was less.

Here, Kelly constructs herself as a good parent by explaining she chose not to deny her son food he enjoys and that this was given priority over portion size. Similarly, Hashani drew on the importance of providing children with food they enjoy when she commented that her daughter's meals are ‘quite samey, but she really likes pizza so it's all right’.

A further way of ensuring children were eating foods they enjoyed was to give them choice over their food consumption. For example, Jane said:

I do tend to give him quite a choice a lot of the time and say ‘Look, we're having this. What do you want?’ Because I think he should have a bit of a choice really because he doesn't have a lot.

Jane's extract suggests that she may have felt guilty about the limited range of food her son could eat, so she compensated for this by allowing him to choose his meals from the available options.

Therefore, at times, parents adjusted the child's food in order to conform to norms relating to portion size and feeding children foods that they enjoy and thus were able to feel they were being good parents. By prioritising these different norms parents were using the child's food as a symbol of their love.

## Discussion

The findings presented above support the fundamental argument in the sociology of food literature that food has intrinsic social functions and meanings attached to it (Beardsworth and Keil 1997, Delormier *et al*. 2009, DeVault 1991). Despite the nature of the ketogenic diet, food was still seen to symbolise inclusion and love. However, this research has also shown that although the meanings attached to foods are ingrained and difficult to challenge, they are not fixed. By coming to view food as medicine parents were able to reverse the negative meanings attached to fat. It could be argued that potentially this was one of the most difficult meanings to alter given the current, and prolonged, emphasis on reducing dietary fat in health promotion campaigns (Blank *et al*. 2009). This adjustment of meaning suggests that the meanings attached to foods are malleable and may be altered if food is used as a medical treatment. As was the case in this study, the meanings attached to food may be more likely to be altered if dietary treatment is successful when previous treatments have had limited efficacy.

It has also been shown that the inherent nature of the ketogenic diet may be problematic for parenting identity. For instance, denying children food (either types or quantities) can lead to parents feeling guilty. However, by being creative with food choice parents were still able to use food to symbolise love and inclusion and construct themselves as good parents. In this sample, success on the diet also contributed significantly to the good parent identity, as these parents felt they had been able to increase their child's quality of life when previous treatments had not been effective. As the views presented above relate only to families who have had success with the diet, additional research is needed to understand the views of parents who find the diet is ineffective and those who choose not to continue implementing it despite efficacy.

It has been illustrated here that the norms relating to child feeding practices do not have a set hierarchy. In this instance norms associated with portion size and the child's enjoyment of food were given priority at different times to justify food choices. This is similar to what Will and Weiner (2014) describe as reconciling repertoires. It has been argued that these norms constitute different repertoires in this context as parents drew on them at different times to justify the choices they had made. Therefore, the norms individuals draw on are likely to be closely linked to the diet under discussion.

It has previously been found that class differences influence parents’ child feeding practices (Backett‐Milburn *et al*. 2006, Wills *et al*. 2011). However, here, although the sample was not particularly diverse, class did not appear to impact on the meanings parents attached to ketogenic foods. One reason for this could be that these parents were united by being able to provide a successful treatment for their child's epilepsy. However, as the participants in this study were predominantly white, mainly from the top quartile of earners and from two‐parent families, additional research would be required to assess whether the findings are applicable to other social groups or family formations. Furthermore, in this article parents have been categorised as a homogenous group because the meanings attached to foods and the identity management techniques used applied to all participants. Any gender differences in the food work taken on by parents will be the focus of another article.

This research has added a new dimension to the literature relating to how families manage dietary change for medical reasons. Previous studies found that when one family member had been recommended dietary alterations for medical reasons, other family members often made the same changes (Gregory 2005, Kelleher 1988, Maclean 1991, Pitchforth *et al*. 2011). Although this study supports this finding to an extent – parents sometimes chose not to eat particular foods in front of the child on the diet – it has also been shown that family members are not always able to adjust their food consumption in line with the recommended diet. Indeed, it was found that when parents were unable to take on the same dietary alterations, many used other techniques to adapt to these changes. For instance, families were able to create ketogenic alternatives of the foods being consumed by others, showing that the diet may be manipulated to fit in with family members’ consumption patterns, as well as others’ food consumption being altered to suit the diet. The ways in which families adapt to dietary changes, therefore, may vary depending on the nature of the diet.

Furthermore, in contrast to Veen *et al*.’s (2013) research, the diet was not demedicalised; in fact, the parents drew heavily on the medical model in their explanations of the diet and food choice. This difference could be explained using Shim's (2010) concept of cultural health capital. Indeed, parents may have developed this way of speaking and thinking about food through their frequent interactions with dieticians, particularly during the first few months of implementing the diet. The frequency with which people interact with dieticians may therefore help to explain the extent to which a diet is medicalised within the family.

Overall, it has been demonstrated that these parents drew on the meanings attached to foods in order to manage their identities. They came to view food as medicine and, as a result, saw fat as good. Their success with this dietary treatment and their ability to improve their child's quality of life overwhelmingly contributed to the good parent identity. The parents also prioritised portion size and the child's enjoyment of food to rationalise their decisions, resulting in parents feeling they were still able to show their child love through the food they provided. Furthermore, they created ketogenic alternatives to include children in social situations, which again enabled them to feel they were being good parents. These important identity management techniques helped these parents to construct themselves as good parents despite the contradictions presented by the diet in relation to feeding norms associated with parenting.

## Note

1 Ketosis is a metabolic state where the body uses ketones rather than glucose for energy.
